# Comprehensive testing of large language models for extraction of structured data in pathology

**DOI:** 10.1038/s43856-025-00808-8

**Published:** 2025-03-31

**Authors:** Bastian Grothey, Jan Odenkirchen, Adnan Brkic, Birgid Schömig-Markiefka, Alexander Quaas, Reinhard Büttner, Yuri Tolkach

**Affiliations:** 1 Institute of Pathology, University Hospital Cologne, Cologne, Germany; 2 Medical Faculty, University of Cologne, Cologne, Germany

**Keywords:** Medical research, Health care, Prostate cancer

## Abstract

**Background:**

Pathology departments generate large volumes of unstructured data as free-text diagnostic reports. Converting these reports into structured formats for analytics or artificial intelligence projects requires substantial manual effort by specialized personnel. While recent studies show promise in using advanced language models for structuring pathology data, they primarily rely on proprietary models, raising cost and privacy concerns. Additionally, important aspects such as prompt engineering and model quantization for deployment on consumer-grade hardware remain unaddressed.

**Methods:**

We created a dataset of 579 annotated pathology reports in German and English versions. Six language models (proprietary: GPT-4; open-source: Llama2 13B, Llama2 70B, Llama3 8B, Llama3 70B, and Qwen2.5 7B) were evaluated for their ability to extract eleven key parameters from these reports. Additionally, we investigated model performance across different prompt engineering strategies and model quantization techniques to assess practical deployment scenarios.

**Results:**

Here we show that open-source language models extract structured data from pathology reports with high precision, matching the accuracy of proprietary GPT-4 model. The precision varies significantly across different models and configurations. These variations depend on specific prompt engineering strategies and quantization methods used during model deployment.

**Conclusions:**

Open-source language models demonstrate comparable performance to proprietary solutions in structuring pathology report data. This finding has significant implications for healthcare institutions seeking cost-effective, privacy-preserving data structuring solutions. The variations in model performance across different configurations provide valuable insights for practical deployment in pathology departments. Our publicly available bilingual dataset serves as both a benchmark and a resource for future research.

## Plain language summary

Pathology departments produce many diagnostic reports as free text, which is hard to analyze or use in research and computer projects. Converting this free text into more standard organized information like test results or diagnoses, makes it easier to use. This task often requires human experts and takes time. Large language models (LLMs), which are advanced computer systems designed to understand and generate human-like text, might simplify this process. Here, we tested six LLMs, including freely available models and the commercial GPT-4 model, using 579 pathology reports in English and German. Our results show that freely available models can perform as well as commercial, providing a cheaper solution while avoiding privacy concerns. The shared dataset will support future research in pathology data processing.

## Abstract (web summary)

Grothey et al. examine the performance of large language models in structuring pathology reports. Findings demonstrate similar accuracy between commercial and open-source models providing a cost-effective, privacy-conscious solution to extract structured data with high precision from bilingual datasets.

## Introduction

**Fig. 1:**
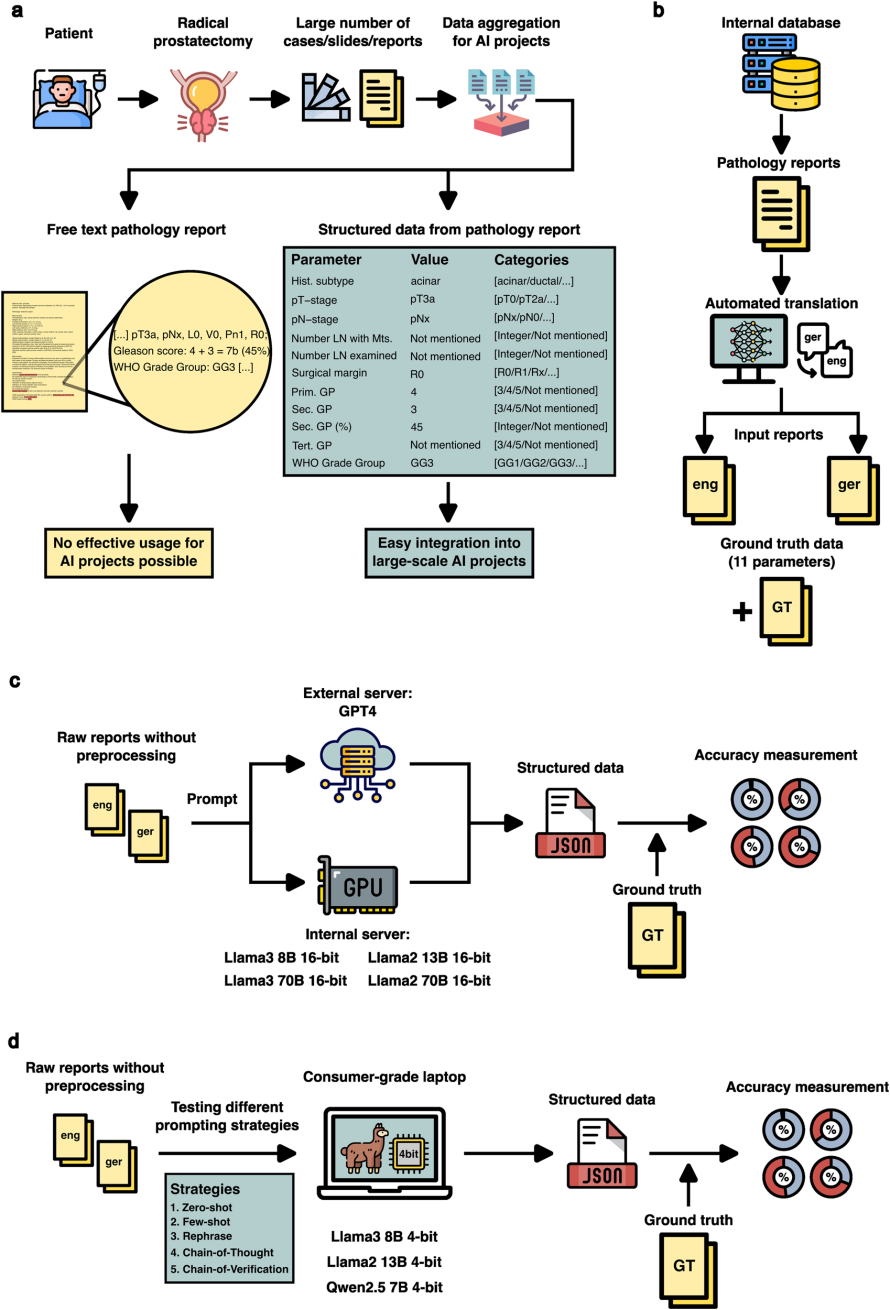
Project overview. **a** Pathology institute archives are valuable data sources for training AI algorithms and gaining insights into disease aspects, especially in oncology. Typically, such archives contain thousands of cases spanning at least the previous 10 years. Pathology reports are crucial for extracting ground truth information for training AI, but only a few departments use structured reporting; most use unstructured, “free text” reports. An example of a pathology report from a patient who underwent radical prostatectomy for prostate cancer is provided, illustrating how data should be structured for AI development. **b** Automated report translation extended the evaluation scope to two languages. 579 radical prostatectomy reports were collected from the institute’s database and translated from German to English using DeepL via API access. **c** First part of the study involved a comparative evaluation of full-weight GPT-4 and 16-bit Llama2/Llama3. Structured data extraction from pathology reports in both German and English was performed for five different LLMs. GPT-4 reports were anonymized and sent to an external OpenAI server via API, while Llama2 and Llama3 were executed on internal infrastructure. The outcomes, structured as JSON files, were analyzed against ground truth data. **d** Second part of the study implemented quantized LLMs and prompting strategies. Since full-weight models require dedicated server hardware, 4-bit quantization allows LLMs to be used directly by end users on consumer-grade laptops, such as MacBook Pro M1 with 16GB RAM. Prompting strategies are essential, user-friendly tools to improve model output. Three different LLMs were tested: Llama2, Llama3, and Qwen2.5.

Pathology departments store a lot of valuable data in their archives. Thus, small and large pathology departments can process between 20,000 and 200,000 cases per year, respectively. Given that the cases (paraffin blocks and histological slides) are archived for at least 10 years, a large pathology institute might possess up to several million unique cases. Depending on specialization, at least 50% of these cases might stem from the oncology domain and represent highly valuable data for the development of pathology AI tools, both diagnostic and advanced (prognostic, predictive), and gaining new insights into different aspects of patient care^1–3^. Systematization, selection, and labeling of these cases for training purposes are possible based on the pathology reports; however, in most departments (at least in Europe), these reports contain free text descriptions and diagnoses^4^. This substantially limits the ability to query the pathology archives for necessary categories of cases and to use these cases for training AI tools that require highly accurate labeling of input data^5,6^ (Fig. 1a). Automated data extraction from pathology reports as using rule-based systems or traditional machine learning approaches previously performed possess limitations in generalizability and high-quality performance^7,8^. These methods often require extensive adaptation for each new application, a time-consuming process that hinders scalability and broad applicability.

Large language models (LLMs) are powerful tools for many applications, including question answering, analyzing and processing texts, reasoning, providing summaries, and insights in the medical domain^9^. Several initial studies investigated the possibility of structured information extraction from both radiology^10–12^ and pathology^13–15^ reports, however, mostly using small datasets, English language reports, and advanced proprietary models (GPT-4, ChatGPT-3.5). The latter are costly, cannot be implemented locally, and are associated with privacy preservation concerns^16^. Many different aspects remain unstudied to date, such as using open-source models, prompt engineering techniques like few-shot or chain-of-thought prompting^17–20^, and quantization of model weights, allowing the models to run locally on consumer-grade hardware (e.g., laptops).

In our study, we create a dataset containing 579 surgical pathology reports, in two versions (English and German languages), with manually obtained high-quality ground truth data for relevant parameters. We study the extraction capabilities of six different models, both proprietary and open-source (GPT4, Llama3 70B, Llama3 8B, Llama2 70B, Llama2 13B, and Qwen2.5 7B) and investigate the influence of prompt engineering and weight quantization on model performance. We show that all relevant parameters can be extracted with high accuracy by both proprietary and open-source models in fully structured forms that can be used for analytical/statistical purposes and downstream AI tool development projects, albeit with substantial differences between the models and setups. This information can be highly valuable for the implementation of LLMs in pathology departments. Moreover, we publicly release the English and German versions of our dataset, which can be used as a research benchmark for further LLMs tests.

## Methods

### Study materials

The study analyzed 579 pathology reports from 340 patients with prostate adenocarcinoma who underwent radical prostatectomy between 2020 and 2022. The reports were retrieved in anonymized plain text format from our institute’s local database. Ground truth data, used to evaluate the model outputs, were manually extracted by a trained doctoral student (human medicine background) from the reports under the supervision of an attending pathologist. Annotation was based on TNM Classification of Malignant Tumors (TNM), Residual tumor (R) classification, Gleason grading system, and WHO tumor classification. Eleven parameters were chosen for the extraction based on their clinical and prognostic relevance: WHO (ISUP) Grade Group, T-Stage, N-Stage, Number of lymph nodes examined, Lymph nodes with metastasis, Resection margins, Histologic subtype, Primary/Secondary/Tertiary Gleason pattern, Percentage of secondary Gleason pattern. These parameters are essential in the staging and characterization of prostate adenocarcinoma as they guide therapeutic decisions and influence patient prognosis.

The pathology reports were translated from German to English (Fig. 1b) using the DeepL API in September 2023 (www.deepl.com). The reports underwent no additional modifications prior to data extraction rather than anonymization.

All study steps were performed in accordance with the Declaration of Helsinki. This study was approved by the Ethical Committee of the University of Cologne (20-1583). The pathological reports used in this study were obtained from patients who signed a broad informed consent (BioMaSOTA consent form), which is routinely collected from patients undergoing therapies at the University Hospital Cologne. This consent allows the use of patient data and biomaterials for academic research and development of commercial products, including data transfer within “Germany, European Union, and outside of European Union (so-called third countries)” (§4 of the consent form). The need for additional patient consent was waived as only anonymized, retrospective materials were used. When transmitting data via API to OpenAI, special attention was paid to ensure full anonymization. The transmitted data was limited exclusively to the pathological-anatomical report with macroscopic and microscopic description, individual summarized results of molecular pathological examinations, and final assessment including TNM classification, making it impossible to trace back to individual patients. Data protection aspects and risks of using cloud-based tools were extensively discussed within authors’ collective.

### Language model hardware, structured data extraction, and analysis workflow

#### Standard model evaluation

Evaluation of GPT-4 and Llama2/Llama3 16-bit precision models (further referred to as full-weight models) ability to extract structured data from pathological reports was performed under various conditions (Fig. 1c) using a dataset of 579 radical prostatectomy pathology reports with English and German versions (see above). Experiments involving GPT-4 utilized the OpenAI API (https://platform.openai.com/). For implementation of Llama2 and Llama3 full-weight models, we used Nvidia A100 80 GB GPUs (CUDA version 117) hosted on our institute’s local infrastructure. The weights for these models were obtained through the Hugging Face API, utilizing the Transformers library in Python^21^ (https://github.com/meta-llama/llama3). Model versions and the access periods are listed in Table 1. We allocated one A100 GPU for the Llama2 13B and Llama3 8B models and four A100 GPUs for the Llama2 70B and Llama3 70B models.

**Table 1:** Large language models specifications and access periods

Model	Version	Access period	Access method	Hardware
GPT-4	gpt-4-1106-preview	January/February 2024	OpenAI API	OpenAI Server
Llama2 13B	Llama-2-13b-chat-hf	January/February 2024	Hugging Face API	1x Nvidia A100 80 GB
Llama2 70B	Llama-2-70b-chat-hf	January/February 2024	Hugging Face API	4x Nvidia A100 80 GB
Llama2 13B 4-bit	llama2:13b	Nov 24	Ollama	MacBook Pro M1 16 GB RAM
Llama3 8B	Meta-Llama-3-8B-Instruct	Apr 24	Hugging Face API	1x Nvidia A100 80 GB
Llama3 70B	Meta-Llama-3-70B-Instruct	Apr 24	Hugging Face API	4x Nvidia A100 80 GB
Llama3 8B 4-bit	llama3:8b	Nov 24	Ollama	MacBook Pro M1 16 GB RAM
Qwen2.5 7B 4-bit	qwen2.5:7b	Nov 24	Ollama	MacBook Pro M1 16 GB RAM

The table lists the model name, version, hardware used, and the period of access. Models include GPT-4, Llama2 variants (13B, 70B, 13B 4-bit), Llama3 variants (8B, 70B, 8B 4-bit), and Qwen2.5 7B 4-bit. GPT-4 was accessed via the OpenAI API. Llama2 and Llama3 full-weight models were hosted on our institute’s local infrastructure using different numbers of Nvidia A100 80 GB GPUs. 4-bit quantized versions of Llama2, Llama3 and Qwen2.5 were hosted on a MacBook Pro M1 with 16 GB RAM using Ollama platform.

Interactions with the language models were performed one report at a time using a zero-shot prompting approach (Supplementary Table S1). The models were tasked to extract eleven key parameters from pathology reports and provide their answers in a structured JSON format (Fig. 1a). The responses generated by the models were automatically evaluated at the case level. 239 patient cases had two or more reports: initial with preliminary data, including preliminary TNM classification, and final that usually incorporated additional investigations such as immunohistochemistry and the information from parallel submissions to the department, e.g., lymphadenectomy specimens, resulting in the final TNM classification. In these cases, priority was given to the most recent report for analysis. If certain parameters were not addressed in the most recent report, subsequent reports were consulted sequentially until the earliest available report. If parameters remained specified as “Not mentioned” in the earliest report, this was considered as the final answer. To evaluate model performance accuracy, precision, recall, and F1-Score were calculated. Accuracy was determined as the ratio of correctly predicted instances to the total number of instances. Precision was calculated as the ratio of true positive predictions to the sum of true positive and false positive predictions. Recall was measured as the ratio of true positive predictions to the sum of true positive and false negatives. F1-Score was computed as the harmonic mean of precision and recall. True positives are the correctly extracted parameters. False positives refer to incorrectly extracted parameters where the model’s output does not match any of the ground truth categories for that parameter (e.g., “pN1” instead of “Pn1”). False negatives refer to incorrectly extracted parameters where the model’s output does exist in the ground truth categories but does not match the specific expected value (e.g., “pN1” instead of “pN3”).

#### Advanced analysis

For detailed error analysis of the full-weight model output, incorrect responses were manually reviewed to identify recurrent error sources.

The frequency of hallucinations was determined by tasking the models with extracting structured data from ten randomly selected, non-malignant, German-language reports using the same zero-shot procedure described above. Since none of these reports contained information on the queried parameters, the only correct answer was “Not mentioned.”

To assess the text complexity within the pathology reports, several metrics were calculated: the number of tokens, type-token ratio (TTR), and measure of textual lexical diversity (MTLD)^22,23^. These calculations were performed using the koRpus package in R. To increase the precision of the complexity measurements, we removed URLs, punctuation, numbers, English stopwords, and whitespaces, and transformed all letters to lowercase. Then, the parameters were evaluated for each individual report. Subsequently, the average values were computed at the patient level. To explore the relationship between text complexity and data extraction accuracy, the average values were correlated with the percentage of correct answers per patient. Correlation coefficients were calculated using Pearson’s r.

#### Quantized models and prompt strategies

For employing the 4-bit quantized versions of Llama2 13B, Llama3 8B and Qwen2.5 7B^24^ models, we used the Ollama platform, executed on a MacBook Pro M1 with 16 GB RAM (https://github.com/ollama). Model versions and the access periods are listed in Table 1. Performance evaluation was conducted as for the full-weight models. Additionally, five most popular and easy to implement alternative prompting strategies (n = 5) were selected as most promising from general language domain and explored during the analysis of quantized versions of the LLMs. To increase the proportion of correctly structured JSON files, we used the built-in JSON mode of the OpenAI API within Ollama. Again, all interactions with the language models were conducted one report at a time.

The zero-shot strategy (Supplementary Table S1) included the pathology report to be analyzed, the parameters to be extracted, the answer options and the output format (JSON file). The few-shot strategy (Supplementary Table S2) is an extension of the zero-shot approach, in which a pathology report and the corresponding JSON output are included as an example to improve performance. For the ‘Rephrase’ strategy (Supplementary Table S3), we tasked GPT-4 to improve the zero-shot prompt we used in previous analyses. In addition, we included several report/JSON examples for GPT-4. Following this, we refined GPT-4’s output by making a single modification before incorporating it into subsequent analyses (see Supplementary Table S3). In the case of the chain-of-thought strategy (Supplementary Table S4), the models were prompted to first break down their response into constituent steps and then summarize them in JSON format. For this purpose, we added the following section at the end of the zero-shot prompt: Walk me through your answer step by step, summarizing and analyzing each category as we go. Then summarize your answer in JSON format at the end. The chain-of-verification strategy (Supplementary Table S5) was a two-step approach to reduce hallucinations by the models. First the models where tasked to extract the structured data using the zero-shot strategy. Then, in a second prompt, they were tasked to compare the output containing the JSON object from the first prompt with the original pathology report again to verify the answers given. The possible response categories were also listed again for this purpose.

### Statistical analysis and graphical visualization

The data processing and statistical analyses were conducted using the R (R Foundation for Statistical Computing, Vienna, Austria) and Python3 programming languages. R was used for the graphical visualization of the results. For further graphical illustrations, Flaticon was used additionally (https://www.flaticon.com).

### Disclosure of artificial intelligence assistance

GPT-4 (Open AI, https://chatgpt.com) was used via the paid chatGPT online platform for language refinements as well as spelling and grammar corrections of the manuscript.

### Reporting summary

Further information on research design is available in the Nature Portfolio Reporting Summary linked to this article.

## Results

### Open-source LLM Llama3 achieves comparable accuracy to GPT-4 in extracting structured data from pathology reports

**Fig. 2:**
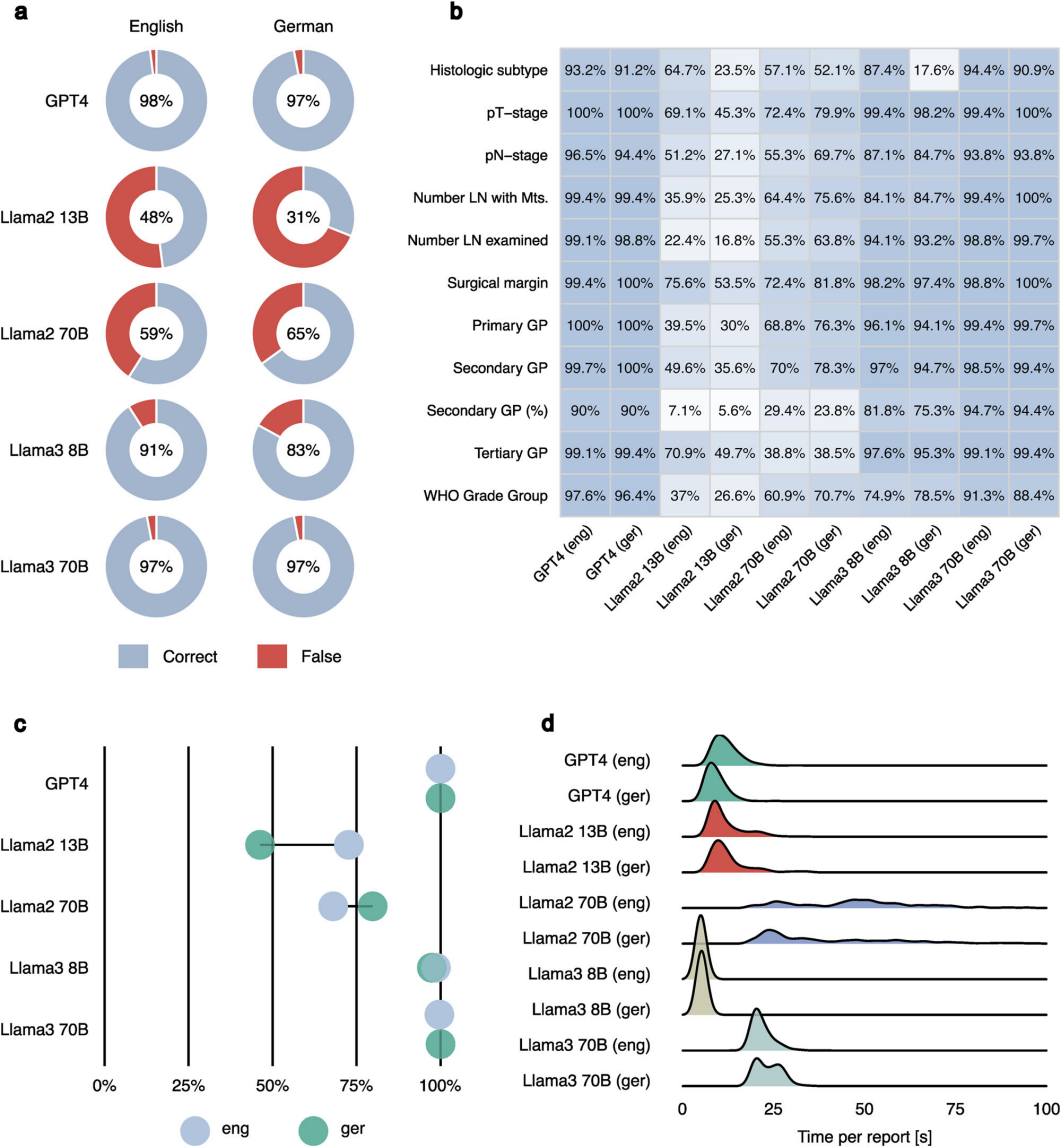
Evaluation of full-weight models. In all figure parts, the evaluation is presented separately for English and German. **a** Overall accuracy by model type: Open-source LLM Llama3 70B achieves accuracy levels equal tonGPT-4. GPT-4 and Llama3 substantially surpass Llama2 in overall accuracy. There are no substantial differences in performance regarding language. Overall accuracy includes all report parameters. **b** Performance evaluation of single report parameters (*n* = 11) extracted in a structured way: Llama3 and GPT-4 achieve consistently robust results with accuracy rates above 90% across all parameters. For Llama2, extracting “Percentage of the secondary Gleason pattern” (Secondary GP (%)) is particularly challenging. GPT-4 exhibits its weakest performance in this category too. In contrast, Llama3 models demonstrate lowest performance for WHO Grade Group parameter. **c** In all tasks, we requested structured data extraction in JSON format, which can be easily used in downstream projects. Percentage of correctly formatted JSON files is presented. Llama2 exhibited shortcomings in generating accurate JSON files for both languages. Llama3 generated incorrectly structured JSON files very rarely. GPT-4 achieved completely error-free formatting. **d** If thousands of reports need processing, runtime is important. Runtime distribution per report is presented for all models. Llama2’s 70B model (local implementation) experienced extended processing times, occasionally exceeding 100 seconds. Processing times for Llama2 13B, GPT-4, and Llama3 models are at a comparable level and appeared competitive. Comment: 21 outliers from Llama2 70B with run times over 100 seconds are not shown. Abbreviations: GP – Gleason Pattern, LN - lymph node, Mts – metastasis, WHO – World Health Organization.

First, we conducted an evaluation of state-of-the-art full-weight proprietary and open- source LLMs focusing on their ability to extract eleven parameters as structured data from pathological reports under various conditions (Fig. 1a, c and methods). GPT-4 and Llama3 70B showed comparable high performance, achieving over 97% overall accuracy (over all parameters) in both languages (Fig. 2a). Notably, good results were also achieved with the much smaller Llama3 8B model (91% and 83% overall accuracy for English and Germany languages, respectively). Conversely, performance of Llama2, previous generation of Llama models, was substantially lower independent on model parameter number (Fig. 2a). Similar trends could be seen, when accuracy was analyzed at the level of single parameters (Fig. 2b). The highest accuracies were observed in parameters related to tumor staging and surgical outcomes, while lower accuracies were in general observed in categories with quantitative parameters (especially for Llama2). GPT-4 and Llama3 70B achieved accuracy rates of at least 88% for each parameter in both languages. Further statistical metrics (Recall, Precision, F1-Score) confirm the trends observed using overall accuracy metric and can be found in Supplementary Table S6 (global level) and Supplementary Data 1 (parameter level).

Importantly, ability to produce a correctly formatted JSON file is a pre-requisite for task completion. We classified the output as a valid JSON file if it was in correct JSON format or if only minimal, automated preprocessing (removing plain text around the brackets) was necessary to achieve it. Invalid JSON outputs commonly included answers given in plain text format without a JSON structure, incorrect usage of brackets, or improperly nested JSON structures. For GPT-4, we observed highly accurate performance by generating entirely valid JSON outputs for all reports and both languages (Fig. 2c). The performance of the Llama3 models was only slightly worse, with nearly flawless results, while Llama2 models experienced difficulties. Given that LLM output is non-deterministic, we repeated the analyses for the open-source models under the same conditions observing almost identical results. Interestingly, a substantial proportion of the invalid JSON files (29% - 100% depending on model and language) in the retest did not match those of the initial run, indicating an intrinsic problem in generating JSON files rather than input-specific issues.

In addition to model task-specific accuracy, the time required for data extraction substantially impacts practical applicability (Fig. 2d). GPT-4 required on average approximately 12 (9) seconds / report for English (German) reports. Notably, Llama3 8B showed the fastest processing times (5-6 seconds/report, both languages). In contrast, Llama2 70B exhibited substantially longer processing times on our hardware, taking an average of 51 (41) seconds for English/German versions. This corrects to 22 (24) seconds for Llama3 70B.

Concluding, we achieved almost error-free data extraction with the GPT-4 and Llama3 70B models. Additionally, we observed a substantial improvement in performance from the Llama2 to the Llama3 model generations.

### Advanced analysis elucidates recurrent sources of error in LLM outputs

**Fig. 3:**
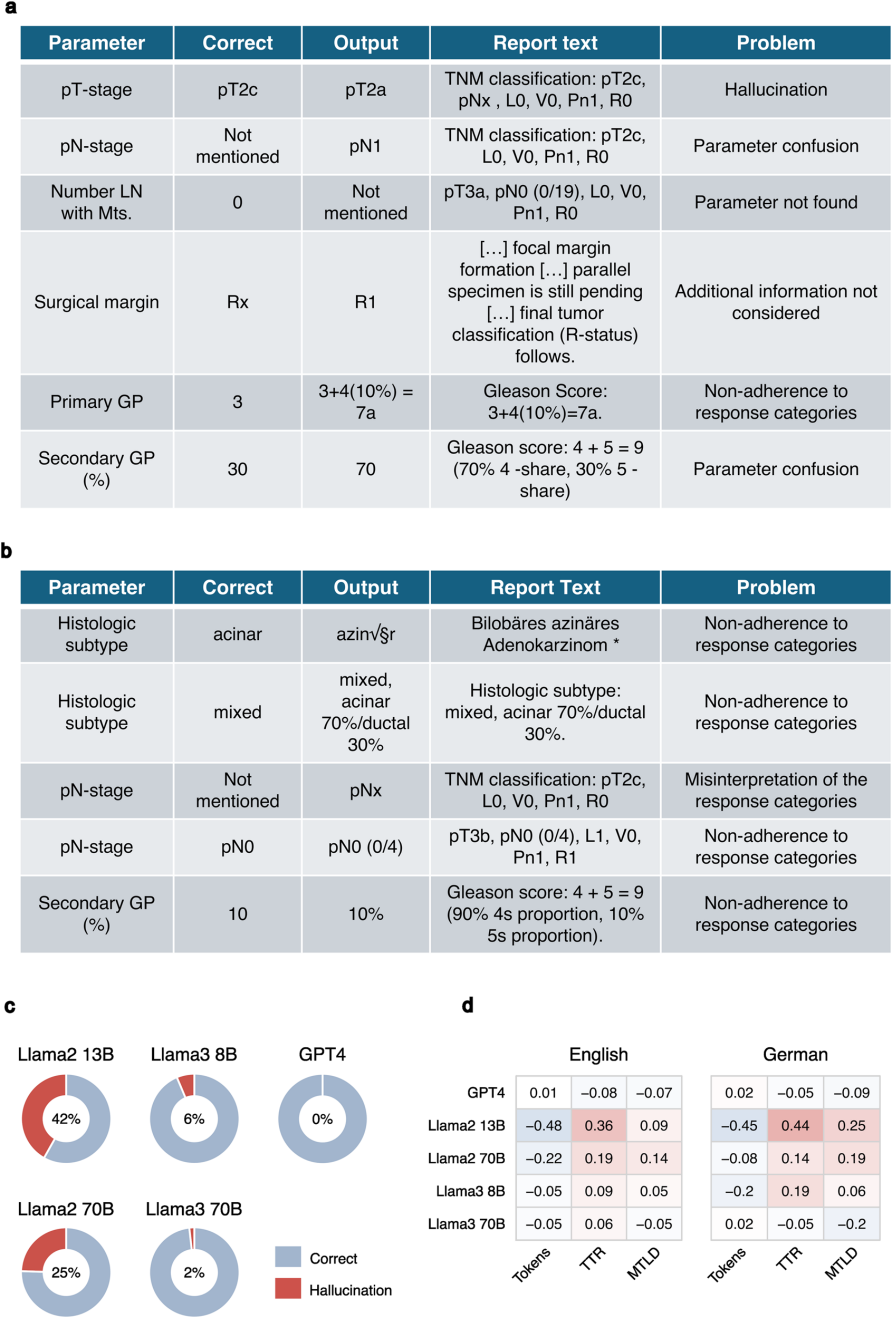
Detailed analysis of the patterns of incorrect responses. **a** Frequently observed fully incorrect model responses and summary of patterns (problem). **b** Partially incorrect model answers: Here, while the general meaning of the answer aligns with the question, adherence to predefined response categories was not maintained. Notably, attempts particularly by Llama2 models to replicate German umlauts (e.g., “azinär” becoming “azin√§r”) introduced additional inaccuracies. (*Translation: Bilobar acinar adenocarcinoma) (**c**) Hallucinations (providing non-existant information) is a common problem in LLMs. Frequency of hallucinations for tested LLM is provided. An additional analysis was conducted whereby the models were instructed to extract structured data from 10 random, non-malignant reports using the same prompt as before. The reports lacked any of the specified parameters, therefore, “Not mentioned” was the correct response. Instances of invalid JSON outputs were subjected to manual examination. A substantial incidence of hallucinatory responses was observed for Llama2 models. GPT4 responses lacked hallucinations with only few reports affected by the problem for Llama3 models. **d** Complexity of the report text can influence the extraction result. We provide case-level accuracy analysis dependent on metrics of lexical diversity (n = 340): Correlation (Pearson’s r) between model accuracies, number of tokens (Tokens), Type-Token Ratio (TTR), and Measure of textual lexical diversity (MTLD). Notably, in some constellations, the Llama2 models exhibit a slight improvement in performance as complexity of the texts increases. In contrast, in case of Llama2 13B, accuracy appears to decrease with an increasing number of tokens, which should be considered in real-world LLM implementation.

After completing the analysis of model performance, we turned to a more detailed analysis of errors found in the LLM output finding some common patterns (Fig. 3a, b) such as confusion between reporting percentages for secondary Gleason patterns and providing the complete Gleason score when only the primary or secondary pattern was requested. Similarly, the models often confused lymph node status (pN) with perineural invasion (Pn) status, especially giving pN1 as an output in cases where pN was not provided at all while perineural invasion (Pn1) was present. Furthermore, we found that the Llama2 models often provided semantically correct responses that did not conform to the pre-specified answer categories (partially incorrect answers, for details see Fig. 3b).

Hallucinations (providing non-existent information) are a common problem of LLMs. In a separate test, we instructed the models to extract structured data from 10 random, non-malignant, German-language reports (Fig. 3c). Since none of these reports contained relevant information, “Not mentioned” was the only correct response. Hallucinations were not observed in GPT-4 responses, which occurred in 2% of responses from Llama3 70B, and in 6% from Llama3 8B. The rates were substantially higher in Llama2 models (Fig. 3c).

Lastly, we analyzed the influence of the text complexity on the extraction result. The following metrics were correlated with extraction accuracy: number of tokens, TTR, and MTLD. Accuracy decreased with the increasing number of tokens for Llama2 (Fig. 3d). Conversely, TTR increases correlated with improved performance, suggesting that higher lexical variety might benefit these models. However, the MTLD, which provides a more accurate measure of lexical diversity, did not exhibit a substantial correlation with model performance. Given the dependency of TTR on token count, we concluded that the variations in model accuracy were primarily influenced by the number of tokens rather than the intrinsic text complexity.

### Quantized LLMs combined with prompting strategies yield heterogeneous results in structured data extraction

**Fig. 4:**
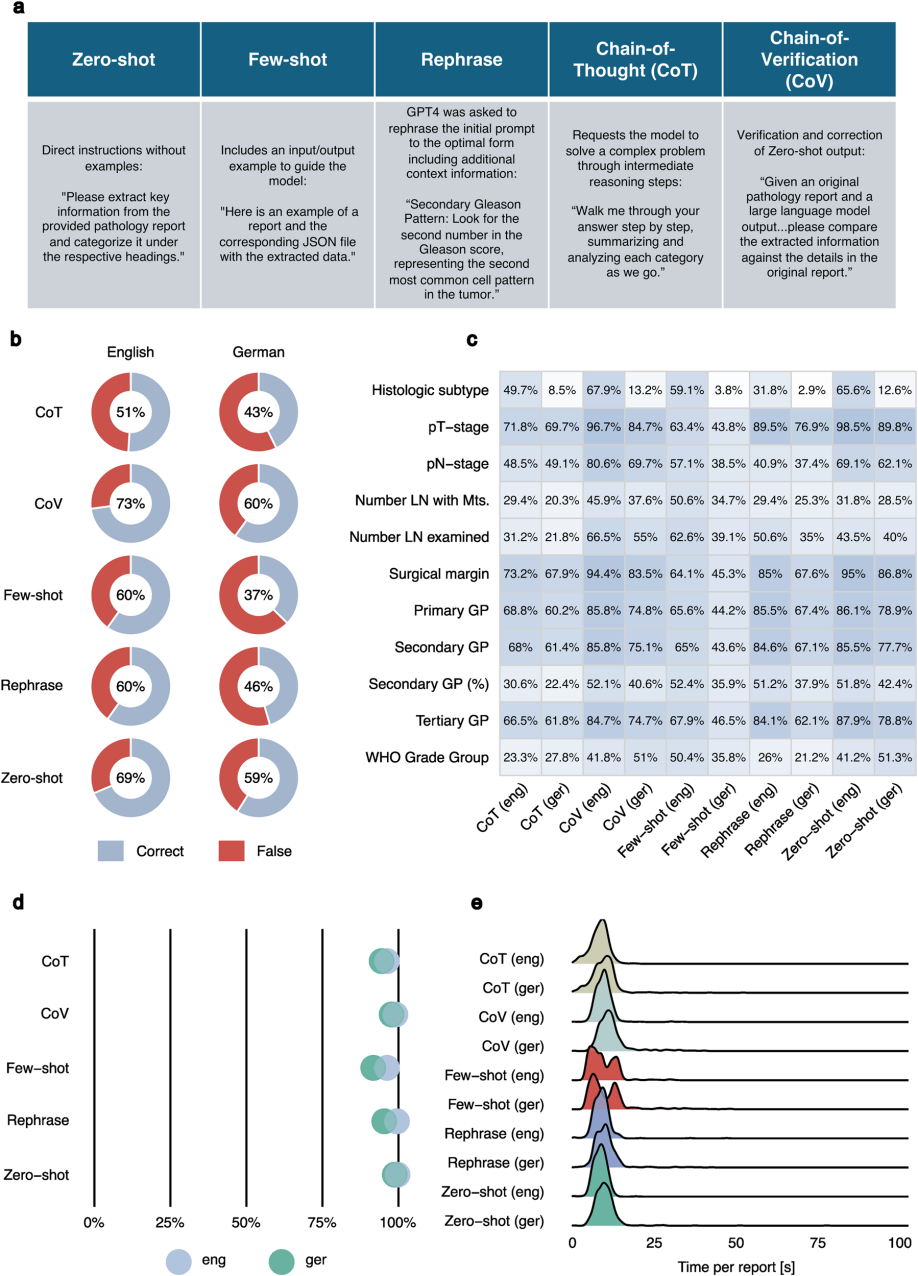
Analysis of accuracy for quantized Llama3 8B 4-bit model with different prompting strategies. Quantization of the model (from 16 bit version to 4-bit version) allows to implement it directly on the consumer-grade laptop. Otherwise, most of the end users will not be able to use the LLMs for report structuring due to the restricted access to specialized AI hardware. As quantization per-definition reduces model performance to some extent, prompting strategies is a logical way to boost the accuracy. **a** Five prompting strategies used in the analysis. Most common and promising prompting strategies from the general domain were selected for implementation in extraction of structured data with 4-bit quantized models. The comparative results are provided for both languages. **b** Overall accuracy for different prompt strategies: Chain-of-verification strategy slightly outperforms all other strategies. **c** Performance evaluation at the level of single report parameters (*n* = 11). **d** Percentage correctly formatted JSON: All prompt strategies produced a high proportion of correct JSON files, with only a few errors, slightly more in the few-shot prompt. Overall, the results for German reports are inferior to those for English reports. **e** Runtime distribution per report: For CoV, only the duration of the secondary analysis (result verification) is accounted for; thus, the initial analysis (zero-shot) time must be additionally considered. One outlier with a run time of over 100 seconds is not shown. Abbreviations: GP Gleason pattern, LN lymph node, Mts metastasis, WHO World Health Organisation), CoT Chain-of-Thought, CoV Chain-of-Verification.

**Fig. 5:**
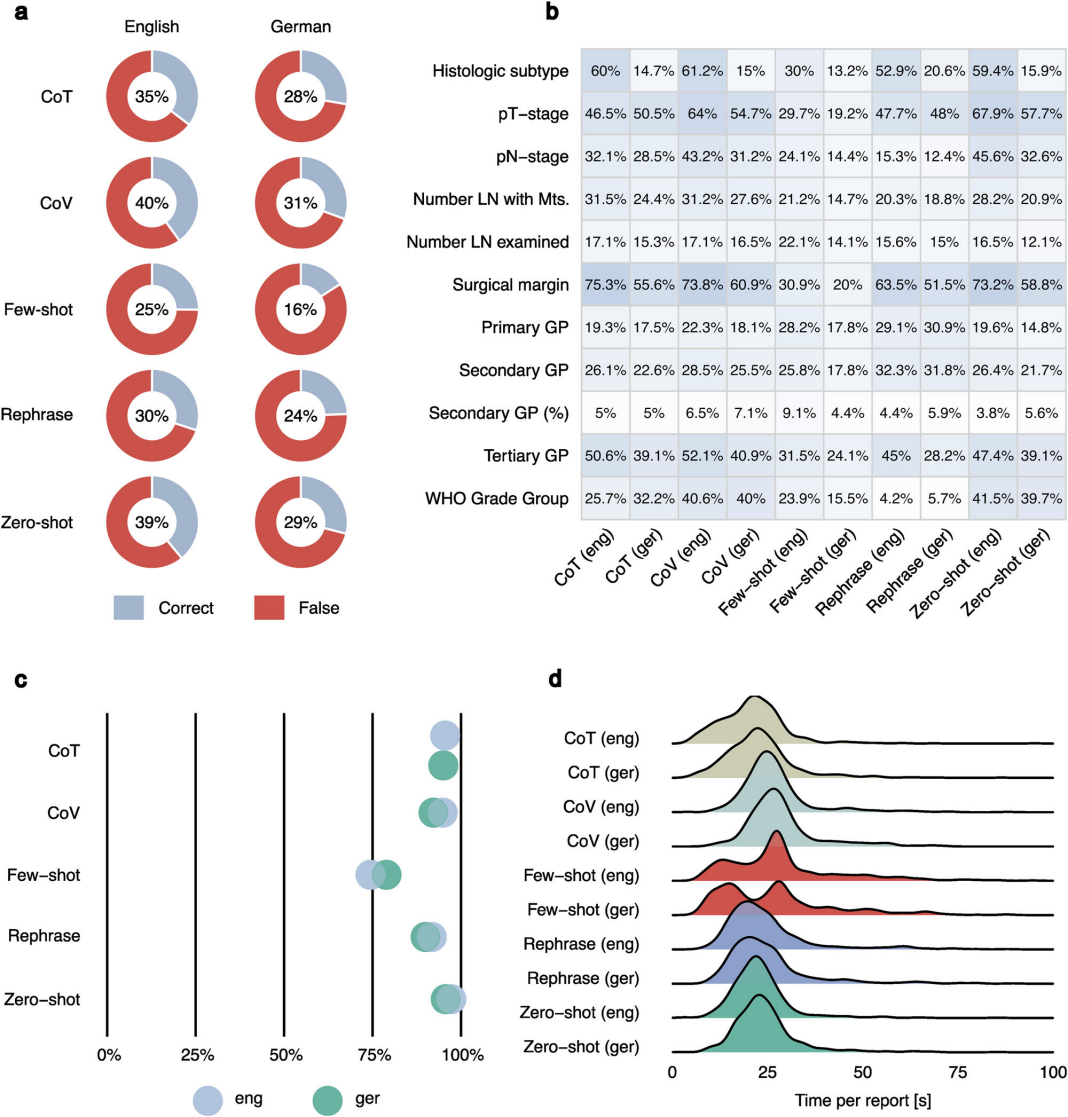
Analysis of accuracy for quantized Llama2 13B 4-bit model with different prompting strategies. **a** Overall accuracy for different prompting strategies: Chain-of-verification prompting consistently outperforms other strategies. **b** Performance evaluation at the level of single report parameters (*n* = 11): Proportion of correct answers averaged for each parameter requested. Heterogeneous results can be observed for the different parameters. **c** Percentage correctly formatted JSON: The results for the few-shot prompting strategy are substantially worse compared to the other strategies. The results of the other strategies are slightly worse or comparable to those of the other two models. **d** Runtime distribution per report: For CoV, only the duration of the secondary analysis (result verification) is accounted for, thus the initial analysis (zero-shot) time must be additionally considered. 75 outliers with run times of over 100 seconds are not shown. Abbreviations: GP Gleason Pattern, LN lymph node, Mts metastasis, WHO World Health Organisation), CoT Chain-of-Thought, CoV Chain-of-Verification.

**Fig. 6:**
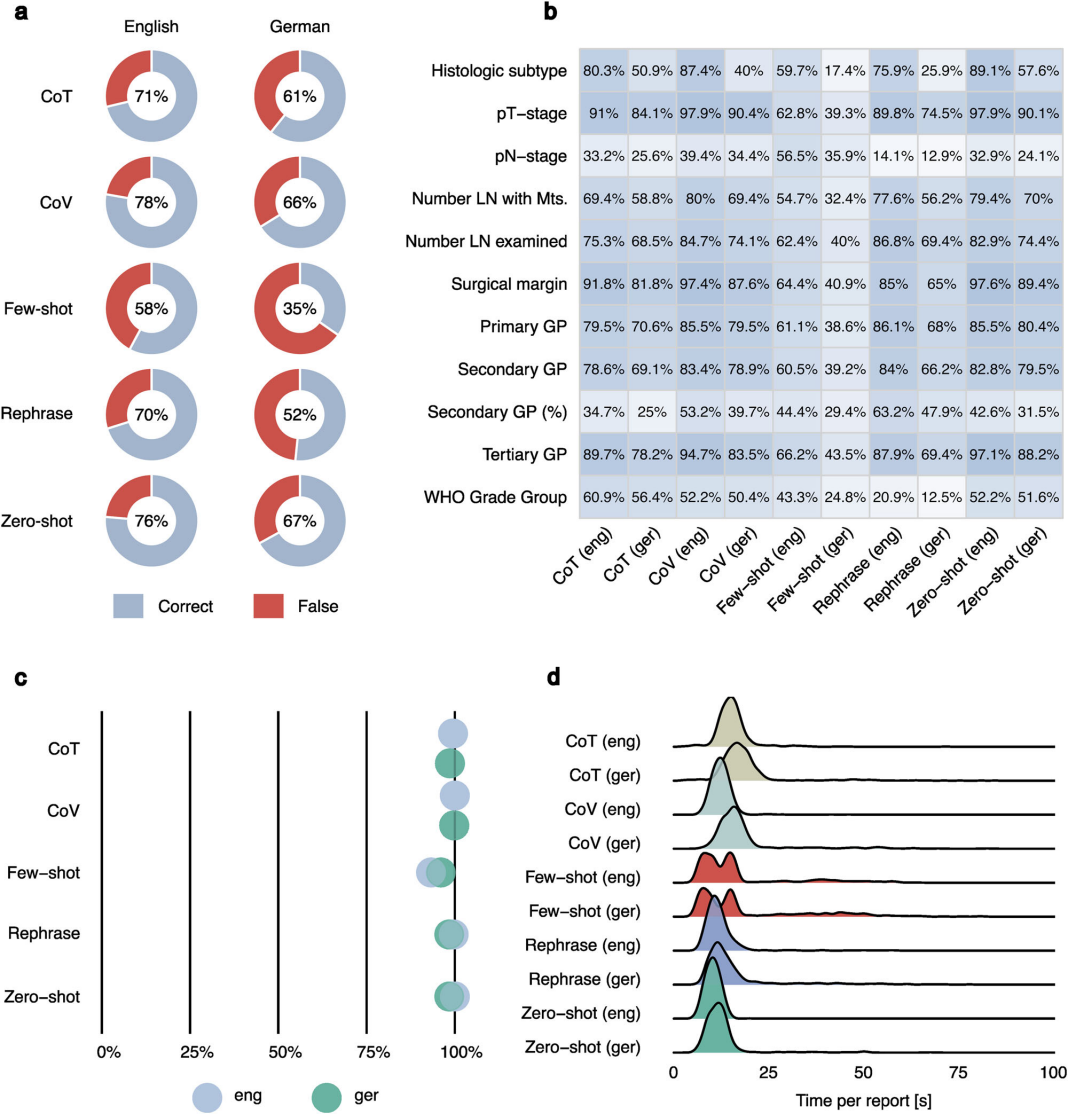
Analysis of accuracy for quantized Qwen2.5 7B 4-bit model with different prompting strategies. **a** Overall accuracy for the different prompting strategies: Zero-shot and chain-of-varification prompts outperform other strategies as well as Llama3 8B 4-bit and Llama2 results. In contrast, few-shot performs substantially worse. **b** Performance evaluation at the level of single report parameters (*n* = 11): Qwen2.5 demonstrates increased performance for most parameters using the CoV or zero-shot prompts. **c** Percentage correctly formatted JSON: In line with previous analyses using the Llama 4-bit model versions, Qwen2.5 encounters some minor challenges when generating JSON files, especially if the few-shot prompt is used. **d** Runtime Distribution per Report: For CoV, only the duration of the secondary analysis (result verification) is accounted for; thus, the initial analysis (zero-shot) time must be additionally considered. Four outliers with run times of over 100 s are not shown. Abbreviations: GP Gleason Pattern, LN lymph node, Mts metastasis, WHO World Health Organisation), CoT Chain-of-Thought, CoV Chain-of-Verification.

Using state-of-the-art open-source models (e.g., Llama2/3) requires high-performance hardware infrastructure, not available to most end users. Quantization (reducing model weight dimensions) is a popular technique allowing LLM deployment on consumer-grade devices (e.g., laptop). In the subsequent section of the study, we explored the efficacy of structured data extraction using quantized open-source LLMs on consumer-grade hardware. Using a typical laptop with GPU support (MacBook Pro M1 with 16GB of RAM) we studied the performance of three open-source LLMs (Llama2 13B, Llama3 8B, Qwen2.5 7B) at 4-bit model weight quantization (Figs. 4–6). In addition to various advancements in Llama model family, several new open-source LLMs independent of Llama technology^24–26^ have been developed. Notably, the Qwen2.5 model has outperformed Llama3 in numerous benchmarks, which is why we included the Qwen2.5 7B 4-bit model in this analysis. Five most popular prompting strategies from the general domain were used to enhance the performance (Fig. 4a). Additionally, to increase the proportion of correctly structured JSON files, we used the built-in JSON mode of the OpenAI API within Ollama. The performance of all three models was substantially reduced (zero-shot implementation) compared to the 16-bit version LLMs tested initially (Figs. 4b, 5a, 6a for Llama3 8B, Llama2 13B, Qwen2.5 7B: zero-shot implementation). Additionally, the accuracy of the different prompting strategies varied considerably. For Llama3 and Llama2, the chain-of-verification strategy yielded the best results (Llama3: English 73%, German 60%; Llama2: English 40%, German 31%). The least effective results within the models were observed using the chain-of-thought (English) or few-shot (German) strategy for Llama3 and the few-shot strategy for Llama2. Qwen2.5 produced the best and most consistent results among the 4-bit models using a chain-of-verification or zero-shot strategy. The general trends in performance for individual report parameters (Figs. 4c, 5b, 6b) aligned closely with those observed in earlier analyses (Fig. 2b, Fig. 5b). Further statistical metrics (Recall, Precision, F1-Score) confirm the evaluation results using overall accuracy metric and can be found in Supplementary Table S7 (global level) and Supplementary Data 2 (parameter level). The analysis concerning percentage of correctly formatted JSON and time per report analysis for comparison with initial results is provided in Figs. 4d, e, 5c, d, and 6c, d.

So far, our findings on structured data extraction using 4-bit quantized models imply that even using state-of-the-art prompting strategies these models do not reach consistent robustness in their outputs. Their performance is by a large margin inferior to that of state-of-the-art LLMs in our analysis (GPT4, Llama3 70B 16 bit).

## Discussion

Our work provides a comprehensive test of how LLMs can be used to fully structure the data from surgical pathology reports in the context of oncological diseases (Fig. 1). Firstly, we create a dataset for resection cases of one type (radical prostatectomy for prostate cancer) with high-quality ground truth information (all relevant tumor parameters manually extracted from all reports in the form of a database; Fig. 1a). This represents a use case generalizable to resection specimens for malignant tumors of other locations as the reports have identical textual structure. Since these reports stem from a German institution, we performed supervised controlled translation into the English language, creating an English copy of the dataset and performing all tests independently for both languages.

Our results show the feasibility of accurately extracting relevant information in a systematized form, independent of the report language. In the best setup, we achieved an impressive overall accuracy of 98% and 97% for English and German languages, respectively (Fig. 2a). For individual parameters (*n* = 11), the range of accuracies was 94-100% for both English and German (Fig. 2b). However, several aspects should be considered to achieve such adequate results.

The type of LLMs used was of utmost importance in our study. We tested six different LLMs: proprietary (GPT4) and open source (Llama3 8B, Llama3 70B, Llama2 13B, Llama2 70B, Qwen2.5 7B), including newly released (April 2024) third generation of Llama models (Llama3). In the classical zero-shot experiment (no special textual prompting of the model and no examples) of the full LLMs, GPT4, Llama3 70B, Llama3 8B, Llama2 70B, Llama2 13B showed overall accuracy of 98%, 97%, 91%, 59%, and 48% (97%, 97%, 83%, 65%, and 31%) for English (German) language (Fig. 2a). There was a clear trend toward lower performance with a lower number of model parameters (a trend known from general applications). Llama2 full-weight models failed to accurately complete the task of generating a JSON file in up to 54% of cases. In contrast, GPT-4, Llama3 70B, and Llama3 7B successfully produced correct JSON files in 100%, 99.8%, and 98% of cases, respectively, even without using a strategy for guaranteed JSON-structured output. We substantially improved Llama2’s performance on this issue for the 4-bit model by leveraging the built-in JSON mode of the OpenAI API in Ollama. Depending on the prompting strategy, the error rate when generating JSON files dropped to a range between 3% to 26%.

Llama3, Llama2, and Qwen2.5 are open-source LLMs, which can be downloaded and used for free on one’s own infrastructure, while GPT4 can only be used via API and is associated with costs (as of January 2024, approximately 0.02 USD/report). Additionally, privacy issues might be a concern when using GPT, as the reports are not anonymized (not the case in our study)^16^. In our work, we showed that open-source LLMs, specifically Llama3, completed the study task with high precision at the same accuracy level as GPT-4, all within a reasonable timeframe suitable for typical clinical applications, even without employing techniques such as batched processing to enhance inference speed. We provide an appealing evidence of how evolution of Llama models extends their capabilities in the medical domain.

Nevertheless, we observed certain patterns of misclassification as summarized in Fig. 3a-d. At least some of the outputs can be treated as partially correct (e.g., pN-Stage: “pN0 (0/4)” instead of “pN0”) and might be addressed through additional post-processing with LLMs; however, they should be acknowledged as mistakes if strict criteria are applied.

Furthermore, we investigated two important aspects intended to democratize the usage of LLMs: prompt engineering and quantization. Prompt engineering (crafting the inquiry to the LLM in a certain way) was shown to substantially improve the accuracy of outputs in general and medical knowledge domain^27^. Numerous well-defined prompting strategies were established^28^. Moreover, quantization of LLM weights (reducing the parameter weight dimensionality, e.g., from 16-bit to 4-bit precision) reduces the size of the model and allows it to run on a consumer-grade laptop. Importantly, we did not study prompt engineering for GPT4 as zero-shot prompting achieved nearly perfect results. Using 4-bit quantized versions of Llama3 8B and Llama2 13B, even with additionally applying JSON mode in Ollama designed to enforce JSON-formatted outputs, overall accuracy substantially declined. For the English (German) dataset, accuracy dropped from 91% and 48% (83% and 31%) with the original 16-bit model versions to 69% and 39% (59% and 29%). Importantly, the application of different prompting strategies mainly led to a decline in accuracy. However, chain-of-verification prompting produced a slight increase in performance.

For the Qwen2.5 model, results showed minor improvements over the Llama3 7B 4-bit model. Similar to the Llama2/Llama3 4-bit model, employing additional prompting strategies did not yield substantial further enhancements. The best accuracy was achieved using zero-shot and chain-of-verification prompting, both demonstrating comparable performance (English: 76% and 78%; German: 67% and 66%)

In conclusion, there is evidence that prompting strategies can substantially influence the performance of even quantized (4 Bit) and small (7B) models. Nevertheless, the performance was, in general, not comparable with GPT4 and Llama3 70B, which provide nearly perfect structuring.

Several studies investigated LLMs for the structuring of pathology report data^13^. Truhn et al.^13^ used GPT4 to process 100 randomly scanned pathology reports in English from The Cancer Genome Atlas colorectal cancer cohort (translated to textual form using optical character recognition), with ground truth manually created. The overall accuracy is comparable to our results. The authors provided limited validation in the German language (report *n* = 21), also achieving 99.4% accuracy for the extraction of parameters. The advantage of our study is a larger dataset (reports *n* = 579) and extensive validation in both English and German languages, generally confirming the high accuracy of GPT4 by Truhn et al. ^13^. Choi et al.^14^ used a dataset of 340 pathology reports from breast cancer merged with ultrasound reports containing text in both Korean and English. For the extraction of single pathological parameters, the authors utilized ChatGPT3.5 (gpt-3.5-turbo) via the GPT for Sheets and Docs tool, achieving an accuracy range of 86.7-96.3%. At that, the extraction accuracy of the “pathological T stage” parameter (one of the most important parameters) was comparably low at 86.7%. However, it provides a good accuracy estimate for the ChatGPT3.5-based pipeline compared to our own and Truhn et al.^13^ results (GPT4-based). Sushil et al.^15^ compared GPT4 and ChatGPT3.5 to supervised methods for 769 breast cancer pathology reports (13 parameters, no TNM-stage; 91.3% in English). The GPT4 model (zero-shot) performed the best with an average macro F1 score of 0.83 compared to 0.75 for the best-supervised model (long-short-term memory with attention) and 0.53 for ChatGPT3.5 (zero-shot). Traditional machine learning methods (e.g., support vector machines, decision trees, gradient boosting) were implemented for extraction of the structured data from pathology reports, however, showing substantially lower accuracy (at least 10%) compared to LLMs^7,8^. This applies to our result achieved by top-performing LLMs when compared to the analysis of Lenain et al.^8^. Moreover, traditional machine learning methods confer several important limitations, such as the necessity of training (difficult or impossible for typical end users), low generalizability to new domains, sensitivity to outliers, and dependence on language and specifically prepared datasets. All these limitations are not applicable to LLMs.

Our study substantially extends beyond the published evidence by evaluating several popular open-source LLMs (Llama3, Llama2, Qwen2.5), which are preferable to GPT4 due to their cost-free nature, substantially mitigated security and privacy concerns resulting from the local running of LLMs on one’s infrastructure, and the ability to run them on consumer-grade laptops in a quantized form. Another study^29^ investigated a very small open-source model, FastChat-T5 (3B parameters), on local infrastructure to process 84 thyroid cancer surgical pathology reports. A substantial limitation of the model is its small context size, which necessitated the conversion of longer text reports to embeddings and measuring similarity between segment embeddings (resulting in two additional analytical steps). Compared to two reviewers for 12 parameters, the average overall accuracy of the model was 89.21%. However, this accuracy pertains to free text formulations and model-generated answers, not to formatting into discrete predefined categories, where the accuracy might be substantially lower.

In this context, the results of our study show that open-source LLMs can also complete this more advanced task with a high degree of precision. Nevertheless, despite the excellent results for some open-source models, especially in the case of the quantized versions, the performance may be further improved by additional fine-tuning (which was out of the scope of this study). Interestingly, in a recent work, Lu et al.^30^ investigated the role of specific medical pretraining for LLMs. The authors show that such medically pretrained models perform better only on tasks where the medical knowledge domain is necessary (e.g., current procedural terminology code classification) but not on more generic tasks, e.g., data extraction (investigated in our study).

Our study is not devoid of limitations. We investigate the performance of LLMs on a large dataset of surgical pathology reports from radical prostatectomies in patients with prostate cancer. Even if the results might be generalizable to other resection specimens of malignant tumors given the high similarity of report structure, this was not explicitly studied in our work. Although we select several of the most popular LLMs (Llama3, Llama2, Qwen2.5), we do show the differences in performance between single open-source models, obviously related to the principles of training (e.g., dataset, fine-tuning, etc.) and the model size. In terms of training-related performance differences, it may also be of interest for future research to use LLMs such as Meditron or Med-PaLM that have been pre-trained on medical data, which we did not investigate in our work. We release English and German versions of our dataset in a fully anonymized form that can be used by research groups for benchmarking other models.

## Conclusions

Our study provides a comprehensive test of LLMs (including common open-source models and the newly released Llama3 family of models) for large-scale extraction of structured information from pathology reports that can be further used for AI algorithm development in the medical domain. We study in detail the prompt engineering and quantization effects on model performance that allow privacy-preserving implementation of the model on a consumer-grade laptop. Our results show that, in addition to multiple advantages (privacy preservation, no processing costs, very low computational profile), open-source models can achieve nearly perfect accuracy levels equal to those of GPT-4 and can be used without any additional supervision, however, not yet in an end user-friendly setup as the performance of quantized model versions is not yet satisfactory. We open-source the large dataset of reports (English and German language) with full ground truth information that can be used as a benchmark for further studies.

## Supplementary information

Supplementary Information

Supplementary Data 1

Supplementary Data 2

Supplementary Data 3

Reporting Summary

## Data Availability

The whole dataset is open-sourced for academic research only on Zenodo^31^. Results data underlying the figures in this publication can be found in Supplementary Data 3. All other data are available from the corresponding authors.

## Code availability

The code underlying this study is available publicly on Zenodo^31^.
