# Supplementary material for: Comprehensive testing of large language models for extraction of structured data in pathology
Source: Commun Med (Lond). 2025 Mar 31;5:96. doi: 10.1038/s43856-025-00808-8 (PMC11958830; doi:10.1038/s43856-025-00808-8)
Supplement: Supplementary file 1 — Supplementary Information [file 43856_2025_808_MOESM1_ESM.pdf]

Supplementary Table S1 Prompting strategies: Zero-shot strategy

Please extract key information from the provided pathology report and categorize it under the respective headings. Listed below are the parameters that should be retrieved from the report. All of them are formatted as follows:

Heading: [Possible options]

Stick precisely to the given answer options for each category. If an answer option specifies 'Integer', ensure the response is provided as an integer value. If the report does not specify information about a particular category or if the information is unclear, provide the response as "Not mentioned."

The output should be structured in a JSON format.

1. WHO (ISUP) Grade Group: [GG1/GG2/GG3/GG4/GG5/Not mentioned]
2. T-Stage (TNM): [pT0/pT2a/pT2b/pT2c/pT3a/pT3b/pT4/Not mentioned]
3. N-Stage (TNM): [pNx/pN0/pN1/Not mentioned]
4. Number of Lymph Nodes examined: [Integer/Not mentioned]
5. Lymph Nodes with Metastasis: [Integer/Not mentioned]
6. Resection Margins: [R0/R1/Rx/Not mentioned]
7. Histologic Subtype: [acinar/ductal/mixed/Not mentioned]
8. Primary Gleason Pattern: [3/4/5/Not mentioned]
9. Secondary Gleason Pattern: [3/4/5/Not mentioned]
10. Tertiary Gleason Pattern: [3/4/5/Not mentioned]
11. Percentage of Secondary Gleason Pattern: [Integer/Not mentioned]

The report reads: [report]

Supplementary Table S2 Prompting strategies: few-shot strategy

Please extract key information from the provided pathology report and categorize it under the respective headings. Listed below are the parameters that should be retrieved from the report. All of them are formatted as follows:

Heading: [Possible options]

Stick precisely to the given answer options for each category. If an answer option specifies 'Integer', ensure the response is provided as an integer value. If the report does not specify information about a particular category or if the information is unclear, provide the response as "Not mentioned."

The output should be structured in a JSON format.

1. WHO (ISUP) Grade Group: [GG1/GG2/GG3/GG4/GG5/Not mentioned]
2. T-Stage (TNM): [pT0/pT2a/pT2b/pT2c/pT3a/pT3b/pT4/Not mentioned]
3. N-Stage (TNM): [pNx/pN0/pN1/Not mentioned]
4. Number of Lymph Nodes examined: [Integer/Not mentioned]
5. Lymph Nodes with Metastasis: [Integer/Not mentioned]
6. Resection Margins: [R0/R1/Rx/Not mentioned]
7. Histologic Subtype: [acinar/ductal/mixed/Not mentioned]
8. Primary Gleason Pattern: [3/4/5/Not mentioned]
9. Secondary Gleason Pattern: [3/4/5/Not mentioned]
10. Tertiary Gleason Pattern: [3/4/5/Not mentioned]
11. Percentage of Secondary Gleason Pattern: [Integer/Not mentioned]

Here is an example of a report and the corresponding JSON file with the extracted data:

Material submitted: Prostate.
Clinical data: Prostate carcinoma, 4/18 biopsies positive, Gleason score 7b.

Pathological-anatomical report:

Macroscopic:
Prostatectomy specimen.
Weight: 58 g.
Prostate: 6.5 x 4 x 3 cm.
Seminal vesicle right: 2.8 x 2.5 x 0.8 cm.

Seminal vesicle left: 3 x 1.8 x 0.7 cm.
Ductus deferens right 2.5 cm long, left 2.8 cm long.
Color markings: ventral midline red, ventral black, dorsal midline green, dorsal blue.
(Apical margin) right (A,B), left (C, D).
(Basal margin of setation) right (E, F), left (G, H).
(Vas deferens margins) right (I), left (J).
(Prostate) Embedding in toto, right half of prostate from apical to basal (K-Z1), left half from apical to basal (Z2-Z18).
(Prostate/Seminal vesicle transition) right (Z19-Z21), left (Z22-Z24).
(Seminal vesicles) right (Z25-Z27), left (Z28-Z31).
(57 Bl, kR).

Microscopic:
In both prostate lobes infiltrates of an acinar differentiated invasive epithelial tumor. Primary architecture: fused glands with partly cribriform growth. Secondary architecture: well-formed glands with a clearly recognizable lumen. Perineural invasion. No intraductal growth. Extraprostatic extension into the adjacent adipose tissue. Tumor-free seminal vesicle and vas deferens. Resection in toto.

Diagnosis:
Bilobar acinar adenocarcinoma of the prostate.
Gleason score: 4 + 3 (20%) = 7b.
WHO grade group: GG3.
Extraprostatic extension.
Perineural invasion.
Tumor-free vas deferens and seminal vesicles.
Resection in toto.

TNM classification (prostate 8th revised edition) (including further submissions):
pT3a, pN1 (2/22), L0, V0, Pn1, R0;

Gleasonscore: 4 + 3 (20%) = 7b.
WHO Grade-group: GG3.

ICD-O-3 Explanation Applies
8140/3 Adenocarcinoma n.d. x
8020/3 Undifferentiated carcinoma n.d.

The corresponding JSON file of the example should have the following format:
{
    "WHO (ISUP) Grade Group": "GG3",
    "T-Stage (TNM)": "pT3a",
    "N-Stage (TNM)": "pN1",
    "Number of Lymph Nodes examined": "22",
    "Lymph Nodes with Metastasis": "2",
    "Resection Margins": "R0",

```
    "Histologic Subtype": "acinar",
    "Primary Gleason Pattern": "4",
    "Secondary Gleason Pattern": "3",
    "Tertiary Gleason Pattern": "Not mentioned",
    "Percentage of Secondary Gleason Pattern": "20"
}
```

The report from which the data needs to be extracted reads: [report]

Supplementary Table S3 Prompting strategies: Rephrase (GPT4)

<u>Strategy:</u>

For the 'Rephrase' strategy, we tasked GPT-4 to improve the Zero-shot prompt we used in previous analyses. Therefore, we used the prompt shown below, which included the Zero-shot prompt and several report/JSON examples. Following this, we refined GPT-4's output by making a single modification before incorporating it into subsequent analyses (Histologic Subtype: […] or “Not mentioned” [Before: …, or not mentioned]).

<u>GPT-4 prompt:</u>

Please extract key information from the provided pathology report and categorize it under the respective headings. Listed below are the parameters that should be retrieved from the report. All of them are formatted as follows:

Heading: [Possible options]

Stick precisely to the given answer options for each category. If an answer option specifies 'Integer', ensure the response is provided as an integer value. If the report does not specify information about a particular category or if the information is unclear, provide the response as "Not mentioned."

The output should be structured in a JSON format.

1. WHO (ISUP) Grade Group: [GG1/GG2/GG3/GG4/GG5/Not mentioned]
2. T-Stage (TNM): [pT0/pT2a/pT2b/pT2c/pT3a/pT3b/pT4/Not mentioned]
3. N-Stage (TNM): [pNx/pN0/pN1/Not mentioned]
4. Number of Lymph Nodes examined: [Integer/Not mentioned]
5. Lymph Nodes with Metastasis: [Integer/Not mentioned]
6. Resection Margins: [R0/R1/Rx/Not mentioned]
7. Histologic Subtype: [acinar/ductal/mixed/Not mentioned]
8. Primary Gleason Pattern: [3/4/5/Not mentioned]
9. Secondary Gleason Pattern: [3/4/5/Not mentioned]
10. Tertiary Gleason Pattern: [3/4/5/Not mentioned]
11. Percentage of Secondary Gleason Pattern: [Integer/Not mentioned]

Given the prompt above, rephrase and expand it to help you do better information extraction from pathology reports (see below for report examples). Maintain all information in the original question.

Here are some report examples and their corresponding JSON file with the extracted data:

Report:
Material submitted: Prostate.
Clinical data: Prostate carcinoma, 4/18 biopsies positive, Gleason score 7b.

Pathological-anatomical report:

Macroscopic:
Prostatectomy specimen.
Weight: 58 g.
Prostate: 6.5 x 4 x 3 cm.
Seminal vesicle right: 2.8 x 2.5 x 0.8 cm.
Seminal vesicle left: 3 x 1.8 x 0.7 cm.
Ductus deferens right 2.5 cm long, left 2.8 cm long.
Color markings: ventral midline red, ventral black, dorsal midline green, dorsal blue.
(Apical margin) right (A,B), left (C, D).
(Basal margin of setation) right (E, F), left (G, H).
(Vas deferens margins) right (I), left (J).
(Prostate) Embedding in toto, right half of prostate from apical to basal (K-Z1), left half from apical to basal (Z2-Z18).
(Prostate/Seminal vesicle transition) right (Z19-Z21), left (Z22-Z24).
(Seminal vesicles) right (Z25-Z27), left (Z28-Z31).
(57 Bl, kR).

Microscopic:
In both prostate lobes infiltrates of an acinar differentiated invasive epithelial tumor. Primary architecture: fused glands with partly cribriform growth. Secondary architecture: well-formed glands with a clearly recognizable lumen. Perineural invasion. No intraductal growth. Extraprostatic extension into the adjacent adipose tissue. Tumor-free seminal vesicle and vas deferens. Resection in toto.

Diagnosis:
Bilobar acinar adenocarcinoma of the prostate.
Gleason score: 4 + 3 (20%) = 7b.
WHO grade group: GG3.
Extraprostatic extension.
Perineural invasion.
Tumor-free vas deferens and seminal vesicles.
Resection in toto.

TNM classification (prostate 8th revised edition) (including further submissions):

pT3a, pN1 (2/22), L0, V0, Pn1, R0;

Gleasonscore: 4 + 3 (20%) = 7b.
WHO Grade-group: GG3.

ICD-O-3 Explanation Applies
8140/3 Adenocarcinoma n.d. x
8020/3 Undifferentiated carcinoma n.d.

JSON file:
{
"WHO (ISUP) Grade Group": "GG3",
"T-Stage (TNM)": "pT3a",
"N-Stage (TNM)": "pN1",
"Number of Lymph Nodes examined": "22",
"Lymph Nodes with Metastasis": "2",
"Resection Margins": "R0",
"Histologic Subtype": "acinar",
"Primary Gleason Pattern": "4",
"Secondary Gleason Pattern": "3",
"Tertiary Gleason Pattern": "Not mentioned",
"Percentage of Secondary Gleason Pattern": "20"
}

Report:
Material sent: prostate.
Clinical data: Prostate carcinoma.

Pathological-anatomical report:

Macroscopic:
55 g prostatectomy specimen measuring 6.5 x 8.2 cm with seminal vesicles attached dorsally on both sides, ductus deferens. Color marking of the prostate ventrally blue, dorsally black.
1st (apex sedimentation margin) Right (A + B), left (C + D) (4 bl, kR).
2nd (bladder floor) deposition right (A + B), left (C + D) (4 bl, kR).
3rd (vas deferens) deposition right (A), left (B) (2 bl, kR).
4th (prostate) lamellation: the cut surface grayish-beige, firm-elastic, fibrous throughout, partly cystic. Embedding in toto, right half of prostate from apical to basal (A - P), left half of prostate from apex to base (Q - Z5) (31 bl, kR).
5. (Seminal vesicles) Right seminal vesicle (A - D), left seminal vesicle (E - H) (8 bl, kR).

Microscopic:
1. fibromuscular tissue with marginal fat and sections of striated muscle and locotypic prostatic glands, some with small prostoliths. No inflammation or atypia.
2. fibromuscular tissue with small focal prostate gland sections without atypia or inflammation.

3. transverse sections through the ductus deferentes with locotypic structure. No inflammation or atypia.
4. standard morphology showing infiltrates of a gland-forming tumor only in the right prostatic lobe. The tumor cells altered with sometimes vigorous prominent nucleoli. The lesion remains confined to the prostate. There is evidence of ingrowth into perineural sheaths. The remaining prostatic tissue shows in part a predominance of fibromuscular portions and in part ectatic, in part atrophic glandular complexes. Included urothelium inconspicuous.
- b.w. -

5. properly built up seminal vesicles on both sides. No evidence of atypia or inflammation.

Tumor blocks for extra examinations: 4A, 4D.

Assessment:
1st-5th prostatectomy specimen with so far only right-sided infiltration by an acinar adenocarcinoma with perineural sheath infiltration.
Tumor-free deposition lines apically and basally. Tumor-free ductus deferentes on both sides. Tumor-free seminal vesicles bilaterally. Residual prostate with adenomyomatosis.

Preliminary TNM classification (Prostate 2017, 8th edition, considering finding C 1211/20): pT2b, pN0 (0/12), L0, V0, Pn1, R0; Gleason score: 3 + 4 = 7a (80% 3's proportion, 20% 4's proportion).

Gleason pattern Gleason score ISUP Graduation group Meets
= 3 + 3 = 6 1
3 + 4 7a 2 x
4 + 3 7b 3
4 + 4 8 4
4 + 5, 5 + 4, 5 + 5 9-10 5

ICD-O-3 Explanation Applies
8140/3 Adenocarcinoma o.n.A. x
8020/3 Undifferentiated carcinoma o.n.A.

Additional examinations are still being performed for verification. Follow-up report will follow.

JSON file:
{
  "WHO (ISUP) Grade Group": "GG2",
  "T-Stage (TNM)": "pT2b",
  "N-Stage (TNM)": "pN0",
  "Number of Lymph Nodes examined": "12",
  "Lymph Nodes with Metastasis": "0",
  "Resection Margins": "R0",
  "Histologic Subtype": "acinar",

"Primary Gleason Pattern": "3",
"Secondary Gleason Pattern": "4",
"Tertiary Gleason Pattern": "Not mentioned",
"Percentage of Secondary Gleason Pattern": "20"
}

Report:
Material sent: prostate.
Clinical data: None.

Pathological-anatomical report:

Macroscopic:
A prostatectomy specimen weighing 44.3 g and measuring 7.2 x 4.2 x 3.2 cm with seminal vesicles and ductus deferentes attached bdsally. Color marking of the prostate ventrally black, dorsally blue.
1. apical margin of deposition, right in (A, B), left in (C, D). (4 bl, kR).
2. base of bladder, right (A, B), left (C, D). (4 bl, kR).
3. sedimentation margins vas deferens, right (A), left (B). (2 bl, kR).
4. lamellation of the prostate. Cut surface, grayish-beige, firm-elastic, fibrous throughout, partly cystic. Embedding in toto, right half of prostate from apical to basal in (A - N), left half from apical to basal in (O - Z3). (29 Bl, kR).
5. embedding of seminal vesicles: Transition of prostate into right seminal vesicle in (A), other parts in (B - G). Transition of the prostate into the left seminal vesicle in (H), further portions in (I - Q). (17 Bl, kR).

Microscopic:
1. fibromuscular tissue with localized prostatic glands. No inflammation or atypia.
2. fibromuscular tissue with loculated prostatic glands. No inflammation or atypia.
3. wall portions of the ductus deferentes with locotypic structure. No inflammation or atypia.
4. infiltrates of a gland-forming tumor in both sides of the prostate (left side significantly more than right side). There is predominantly a confluent growth pattern showing single glomeruloid sections. Occasional retraction artifacts. Prominent nucleoli in the nuclei. Quite circumscribed solid nodules as well.
- b.w. -

Perineural sheath infiltrations again and again. The ink-marked preparation margin dorsal left is partially infiltrated to 1 - 2 mm. No overlapping prostate tissue. Residual prostate with partly predominant fibromuscular parts, partly indicated nodular structure.
5. seminal vesicle tissue on both sides with locotypical structure. No inflammation or atypia. Partly still included prostate tissue on the left side with further infiltrates of the previously described tumor.

Tumor blocks for extra examinations: 4 H, 4 P, 4 R.

Assessment:
1st-5th prostatectomy specimen with bilateral infiltrates of acinar adenocarcinoma and perineural sheath infiltration. Marginal formation dorsal left midline, maximum 2 mm. Tumor-free deposition lines apically and basally. Tumor-free ductus deferens settling lines. Tumor-free seminal vesicles on both sides.

TNM classification (Prostate 2017, 8th edition): pT2c, L0, V0, Pn1, R1;

Gleason score: 4 + 3 = 7b (60%4share, 35%4share, 35%3share); tertiary grade: 5 (5%).

Gleason pattern Gleason score ISUP Graduation group Meets
= 3 + 3 = 6 1
3 + 4 7a 2
4 + 3 7b 3 x
4 + 4 8 4
4 + 5, 5 + 4, 5 + 5 9-10 5

R1 situation:
Infiltrated resection margin Gleason pattern Extent of margin infiltration (mm)
dorsal center left 3 maximum 2

ICD-O-3 Explanation Applies
8140/3 Adenocarcinoma n.d. x
8020/3 Undifferentiated carcinoma o.n.A.

For verification, we will still perform additional examinations and report subsequently.

JSON file:

```
{
  "WHO (ISUP) Grade Group": "GG3",
  "T-Stage (TNM)": "pT2c",
  "N-Stage (TNM)": "Not mentioned",
  "Number of Lymph Nodes examined": "Not mentioned",
  "Lymph Nodes with Metastasis": "Not mentioned",
  "Resection Margins": "R1",
  "Histologic Subtype": "acinar",
  "Primary Gleason Pattern": "4",
  "Secondary Gleason Pattern": "3",
  "Tertiary Gleason Pattern": "5",
  "Percentage of Secondary Gleason Pattern": "35"
}
```

Report:
Sent material: 1st LK left. 2. LK right. 3. periprostatic adipose tissue. 4. prostate.
5. seminal vesicle on the left.
Clinical data:

Pathologic anatomic report:

Macroscopically:
1. (LK left) 7 x 5 x 2 cm measuring tissue with nodular condensations. A nodule measuring max 1.5 cm bisected in (A+B), a split nodule measuring max 4 cm in (C-F), a split nodule in each (G+H), further nodular condensations in (I-M) (13 Bl, R).
2nd (LK right) 6.5 x 5 x 1.5 cm measuring lipomatous tissue with nodular consolidations. One cleft nodule in (A+B), another cleft nodule in (C+D), more nodular consolidations in (E-J) (10 Bl, R).
3rd (Periprostatic fat) lipomatous tissue measuring 3.5 x 1.5 x 0.5 cm. In toto in (1 bl, kR).
4. (Prostate) Prostatectomy, bds. dorsal seminal vesicles and ductus deferentes.
Prostate dimensions: left/right 5.2 cm, ventral/dorsal 3.8 cm, basal/apical 4.5 cm.
The left seminal vesicle not included on the main specimen, the right seminal vesicle
4.5 x 1.5 x 0.5 cm.
Left ductus deferens: 4.5 cm long and 0.6 cm in diameter.
Right deferent duct: 5.5 cm long and 0.6 cm in diameter.

Color marking of the prostate: clinically already inked.
Ventral: green, ventral midventral red, dorsal blue, dorsal midventral black.
Seminal vesicles: blue.

(Apical sedimentation margin) Right in (A, B), left in (C, D).
(Basal sedimentation margin) Right (E, F), left (G, H).
(Sedimentation margins vas deferens) Right (I), left (J).

- b.w. -

(prostate) embedding in toto, right half of prostate from apical to basal according to scheme in (K-Z12), left half from apical to basal according to scheme in (Z13-Z39).
(Transition prostate to right seminal vesicle) (Z40-Z42).
(Transition prostate/left ductus deferens) (Z43-Z45).
(Seminal vesicles) right seminal vesicle in (Z46-Z51). Left ductus deferens in toto in (Z52+Z53) (70 Bl, kR).
5th (left seminal vesicle) seminal vesicle resectate measuring 4 x 1.7 x 0.7 cm. The surface is inked in blue. In toto in (A-D). (4 bl, kR).

Microscopic:
1st + 2nd Regularly structured encapsulated lymph nodes with regular lymphoid follicle formation adjacent to mature adipose tissue. No atypical cell infiltrates.
3. vascular and nerve-guiding mature adipose tissue. No atypical cells.
4. prostatic parenchyma with infiltrates by a malignant gland-forming tumor. The glands are predominantly back-to-back and partly confluent. Nuclei somewhat pleomorphic and enlarged with finely granulating chromatin pattern and prominent nucleoli. Perineural sheath infiltration. No blood vessel or lymphatic vessel invasion.
Regularly structured seminal vesicle. No atypical cell infiltrates.
5. Regularly structured seminal vesicle. No atypical cell infiltrates.

Assessment:
1. eleven tumor-free lymph nodes (0/11) (lymph nodes on the left).
2. ten tumor-free lymph nodes (0/10) (lymph nodes on the right).
3. tumor-free periprostatic fat tissue.
4. prostatectomy specimen showing infiltrates of both halves of the prostate by acinar adenocarcinoma of the prostate with perineural sheath infiltration. No blood or lymphatic vessel invasion.
Tumor-free apical and basal resection margins on both sides.
Tumor-free vas deferens resection margins bilaterally.
Tumor-free and structurally intact seminal vesicle on the right.
5. tumor-free and structurally intact seminal vesicle on the left.

TNM classification (Prostate 2020 8th revised edition): pT2c, pN0 (0/21), L0, V0, Pn1, R0;
Gleason score: 3 + 4 = 7a (60% 3s proportion, 40% 4s proportion);
Gleason pattern Gleason score ISUP Graduation group Meets
= 3 + 3 = 6 1
3 + 4 7a 2 x
4 + 3 7b 3
4 + 4 8 4
4 + 5, 5 + 4, 5 + 5 9-10 5

ISUP grading 2.
ICD-O-3 Explanation Applies
8140/3 Adenocarcinoma o.n.a. x
8020/3 Undifferentiated carcinoma o.n.A.

JSON file:
{
  "WHO (ISUP) Grade Group": "GG2",
  "T-Stage (TNM)": "pT2c",
  "N-Stage (TNM)": "pN0",
  "Number of Lymph Nodes examined": "21",
  "Lymph Nodes with Metastasis": "0",
  "Resection Margins": "R0",
  "Histologic Subtype": "acinar",
  "Primary Gleason Pattern": "3",

  "Secondary Gleason Pattern": "4",
  "Tertiary Gleason Pattern": "Not mentioned",
  "Percentage of Secondary Gleason Pattern": "40"
}

Report:
1. supplementary report from 22.09.2021

Pathological-anatomical report:

Pending were supplemental immunohistochemical studies to further characterize the carcinoma.
(4x IHC, 1x HE).
Tumor cells show preserved expression of ATM, PTEN, MSH6, PMS2.

Assessment:
Bilobar adenocarcinoma of the prostate.
Histological subtype: acinar.
Gleason score: 3+4(40%)=7a.
WHO Grade Group: GG 2.
Perineural sheath infiltration.
No extraprostatic extension.
No infiltration of the urinary bladder neck muscles.

No intraductal extension.
No angioinvasion.
No seminal vesicle infiltration.

Resection in sano.
Tumor-free margins of both vas deferens and both seminal vesicles.

TNM classification (Prostate 2020 8th revised edition).
With consideration of parallel findings:
pT2c, pN0 (0/17), L0, V0, Pn1, R0;
Gleason score: 3+4(40%)=7a.
WHO Grade Group: GG 2.

ICD-O-3 Explanation Applies
8140/3 Adenocarcinoma o.n.A. x
8020/3 Undifferentiated carcinoma o.n.A.

Comment:
No immunohistochemical correlate for PTEN deletion.

MSI status: stable, MSS (immunohistochemistry).

Pending further complementary studies to characterize the carcinoma (PanelSeq),

a follow-up report will follow.

JSON file:
{
  "WHO (ISUP) Grade Group": "GG2",
  "T-Stage (TNM)": "pT2c",
  "N-Stage (TNM)": "pN0",
  "Number of Lymph Nodes examined": "17",
  "Lymph Nodes with Metastasis": "0",
  "Resection Margins": "R0",
  "Histologic Subtype": "acinar",
  "Primary Gleason Pattern": "3",
  "Secondary Gleason Pattern": "4",
  "Tertiary Gleason Pattern": "Not mentioned",
  "Percentage of Secondary Gleason Pattern": "40"
}

Report:
Material sent: prostate.
Clinical data: Prostate carcinoma, intermediate risk, Gleason 7b. 5/6 punches positive; iPSA 12.8. tumor stage?

Pathologic Anatomic Report:

Macroscopic:
(prostate) prostatectomy, bds. dorsal seminal vesicles and ductus deferentes.
Weight: 48 g.
Prostate dimensions: 5.2 x 4.8 x 4.7 cm.
Left seminal vesicle: 4.3 x 3.2 x 2.2 cm.
Right seminal vesicle: 3.7 x 3.0 x 1.5 cm.
Left ductus deferens: 4.5 cm long.
Right deferent duct: 4.2 cm long.

Color marking of prostate: ventral: black, ventral midline: red, dorsal: blue, dorsal midline: green, seminal vesicles: blue.
In the base area centrally seated two nodes measuring 1.5 and 1.2 cm each.

(Apical settling margin) Right in (A, B), left in (C, D).
(Basal sedimentation margin) Right (E-I), left (J-L).
(Sedimentation margins vas deferens) Right (I), left (J).
(Prostate) Embedding in toto, right half of prostate from apical to basal according to scheme in (M-V), left half from apical to basal according to scheme in (W-Z7).
(transition prostate/ seminal vesicle) right (Z8-Z14), left (Z15-Z20).
(seminal vesicle deposition) Right seminal vesicle in (Z21) Left seminal vesicle in (Z22)

(Seminal vesicles, vas deferens) Right in (Z23 -Z29), left in (Z30-Z34) (60 Bl, kR).

- b.w. -

Microscopic:
Infiltrates of acinar differentiated adenocarcinoma are seen in embeddings from both lobes of the prostate. Primary architectural pattern: fused, poorly formed glands, suggested cribriform complexes (small cribriform). No larger cribriform complexes. No definite intraductal spread. Perineural sheath invasion. Periprostatic spread with widespread infiltration of seminal vesicles. Infiltration of the urinary bladder neck muscles. No definite angioinvasion.

Assessment:
Bilobaric acinar adenocarcinoma of the prostate.
Gleason score: 4+3 (20%) = 7b.
WHO Grade Group: GG3.
Perineural sheath invasion.
No definite angioinvasion.
Extraprostatic extension with widespread infiltration of both seminal vesicles.
Infiltration of the urinary bladder neck muscles.
No intraductal extension.
Resection in sano.
Tumor-free are sedimentation margins of both vas deferens.

TNM classification (Prostate 2020 8th revised edition): pT3b, pNx, L0, V0, Pn0, R0.

Gleason score: 4 + 3 (20%) = 7b.
WHO Grade Group: GG3.

ICD-O-3 Explanation Applies
8140/3 Adenocarcinoma o.n.A. x
8020/3 Undifferentiated carcinoma o.n.A.

Comment:
Pending are individual immunohistochemical studies to further characterize the carcinoma as well as the review of the parallel submission.
A follow-up report will follow.

JSON file:
{
  "WHO (ISUP) Grade Group": "GG3",
  "T-Stage (TNM)": "pT3b",
  "N-Stage (TNM)": "pNx",
  "Number of Lymph Nodes examined": "Not mentioned",

"Lymph Nodes with Metastasis": "Not mentioned",
"Resection Margins": "R0",
"Histologic Subtype": "acinar",
"Primary Gleason Pattern": "4",
"Secondary Gleason Pattern": "3",
"Tertiary Gleason Pattern": "Not mentioned",
"Percentage of Secondary Gleason Pattern": "20"
}

GPT-4 answer:

When reviewing a pathology report, your task is to meticulously extract specific information related to the prostate cancer diagnosis and categorize this information under predefined headings. Each category has a set of possible options from which you must choose the most appropriate based on the report's contents. If the report does not mention a particular category or if the information is ambiguous, you should mark it as "Not mentioned." Below are the categories and their possible options, along with additional context to guide your extraction process:

WHO (ISUP) Grade Group: This refers to the grading of the prostate cancer based on the Gleason scoring system, revised into the International Society of Urological Pathology (ISUP) grade groups. Look for mentions of the Gleason score and its conversion into a Grade Group (GG1 to GG5).
T-Stage (TNM): The Tumor stage according to the TNM (Tumor, Node, Metastasis) classification system. It describes the size and extent of the main tumor. T-stages specifically for prostate cancer range from pT0 to pT4.
N-Stage (TNM): This category describes whether the cancer has spread to nearby lymph nodes and, if so, how many. Options include pNx (nodes were not assessed), pN0 (no cancer in nearby nodes), and pN1 (cancer has spread to nearby nodes).
Number of Lymph Nodes examined: Provide the number of lymph nodes that were surgically removed and examined under a microscope. If this number is not specified, mark as "Not mentioned."
Lymph Nodes with Metastasis: Indicate the number of lymph nodes that were found to contain cancer. If not mentioned, mark as "Not mentioned."
Resection Margins: This indicates whether cancer cells were found at the edge of the removed tissue, suggesting that some cancer might have been left behind. Options include R0 (no cancer at margins), R1 (cancer at the margins), and Rx (cannot be assessed).
Histologic Subtype: Identifies the type of cells that the cancer started in. Options include acinar, ductal, mixed, or “Not mentioned”.
Primary Gleason Pattern: Look for the first number in the Gleason score, which represents the most common pattern of cells found in the tumor.
Secondary Gleason Pattern: Look for the second number in the Gleason score, representing the second most common cell pattern in the tumor.
Tertiary Gleason Pattern: If mentioned, this is the third pattern observed in less common instances within the tumor. If not specified, mark as "Not mentioned."

Percentage of Secondary Gleason Pattern: This refers to the proportion (as a percentage) of the tumor that consists of the secondary Gleason pattern. Provide this number as an integer or mark as "Not mentioned" if unspecified.

For each report, create a JSON object with the above categories as keys and the extracted information as values. Be meticulous in reading the report to ensure accuracy, especially in identifying the correct Gleason patterns and their percentages, as well as the precise staging information according to the TNM classification.

The report reads: [report]

Supplementary Table S4 Prompting strategies: Chain-of-Thought strategy

Please extract key information from the provided pathology report and categorize it under the respective headings. Listed below are the parameters that should be retrieved from the report. All of them are formatted as follows:

Heading: [Possible options]

Stick precisely to the given answer options for each category. If an answer option specifies 'Integer', ensure the response is provided as an integer value. If the report does not specify information about a particular category or if the information is unclear, provide the response as "Not mentioned."

1. WHO (ISUP) Grade Group: [GG1/GG2/GG3/GG4/GG5/Not mentioned]
2. T-Stage (TNM): [pT0/pT2a/pT2b/pT2c/pT3a/pT3b/pT4/Not mentioned]
3. N-Stage (TNM): [pNx/pN0/pN1/Not mentioned]
4. Number of Lymph Nodes examined: [Integer/Not mentioned]
5. Lymph Nodes with Metastasis: [Integer/Not mentioned]
6. Resection Margins: [R0/R1/Rx/Not mentioned]
7. Histologic Subtype: [acinar/ductal/mixed/Not mentioned]
8. Primary Gleason Pattern: [3/4/5/Not mentioned]
9. Secondary Gleason Pattern: [3/4/5/Not mentioned]
10. Tertiary Gleason Pattern: [3/4/5/Not mentioned]
11. Percentage of Secondary Gleason Pattern: [Integer/Not mentioned]

Walk me through your answer step by step, summarizing and analyzing each category as we go. Then summarize your answer in JSON format at the end.

The report reads: [report]

Supplementary Table S5 Prompting strategies: Chain-of-Verification strategy

Given an original pathology report and a large language model output containing a JSON object with key information extracted from this report, please compare the extracted information against the details in the original report. Your task is to verify the accuracy of the extracted information and ensure its completeness based on the provided categories. Each category has specific response options that you must strictly follow. If any discrepancies or omissions are found, correct the JSON object accordingly. The categories and their response options are as follows:

1. WHO (ISUP) Grade Group: [GG1/GG2/GG3/GG4/GG5/Not mentioned]
2. T-Stage (TNM): [pT0/pT2a/pT2b/pT2c/pT3a/pT3b/pT4/Not mentioned]
3. N-Stage (TNM): [pNx/pN0/pN1/Not mentioned]
4. Number of Lymph Nodes examined: [Integer/Not mentioned]
5. Lymph Nodes with Metastasis: [Integer/Not mentioned]
6. Resection Margins: [R0/R1/Rx/Not mentioned]
7. Histologic Subtype: [acinar/ductal/mixed/Not mentioned]
8. Primary Gleason Pattern: [3/4/5/Not mentioned]
9. Secondary Gleason Pattern: [3/4/5/Not mentioned]
10. Tertiary Gleason Pattern: [3/4/5/Not mentioned]
11. Percentage of Secondary Gleason Pattern: [Integer/Not mentioned]

Ensure that each field adheres to the specified options. If an answer option specifies 'Integer', ensure the response is provided as an integer value. If a category is not mentioned in the original report, mark it as "Not mentioned". Do not add or infer information that is not explicitly stated in the report.

The report reads:

[report]

The large language model output containing the JSON object reads:

[Zero-shot JSON-file]

Your answer should be structured in JSON format again.

Supplementary Table S6: Recall, Precision and F1–Score for full–weight LLMs (global level)

	Model	Precision	Recall	F1_Score
1	gpt4_eng	0.99	0.98	0.99
2	gpt4_ger	0.99	0.97	0.98
3	llama2_13b_eng	0.73	0.57	0.64
4	llama2_13b_ger	0.66	0.36	0.47
5	llama2_70b_eng	0.85	0.65	0.74
6	llama2_70b_ger	0.86	0.72	0.78
7	llama3_70b_eng	0.99	0.98	0.98
8	llama3_70b_ger	0.98	0.98	0.98
9	llama3_8b_eng	0.94	0.95	0.95
10	llama3_8b_ger	0.87	0.94	0.90

Supplementary Table S7: Recall, Precision and F1–Score for quantized LLMs and different prompting strategies (global level)

	Model_Strategy	Precision	Recall	F1_Score
1	llama2_13b_4bit_cot_eng	0.44	0.65	0.52
2	llama2_13b_4bit_cot_ger	0.33	0.64	0.43
3	llama2_13b_4bit_cov_eng	0.49	0.67	0.57
4	llama2_13b_4bit_cov_ger	0.38	0.62	0.47
5	llama2_13b_4bit_fewshot_eng	0.34	0.48	0.40
6	llama2_13b_4bit_fewshot_ger	0.20	0.43	0.27
7	llama2_13b_4bit_rephrase_eng	0.37	0.61	0.46
8	llama2_13b_4bit_rephrase_ger	0.29	0.60	0.39
9	llama2_13b_4bit_zeroshot_eng	0.47	0.69	0.56
10	llama2_13b_4bit_zeroshot_ger	0.34	0.65	0.45
11	llama3_8b_4bit_cot_eng	0.59	0.79	0.67
12	llama3_8b_4bit_cot_ger	0.51	0.72	0.60
13	llama3_8b_4bit_cov_eng	0.81	0.87	0.84
14	llama3_8b_4bit_cov_ger	0.67	0.85	0.75
15	llama3_8b_4bit_fewshot_eng	0.65	0.87	0.75
16	llama3_8b_4bit_fewshot_ger	0.42	0.77	0.54
17	llama3_8b_4bit_rephrase_eng	0.67	0.84	0.75
18	llama3_8b_4bit_rephrase_ger	0.53	0.76	0.62
19	llama3_8b_4bit_zeroshot_eng	0.78	0.85	0.81
20	llama3_8b_4bit_zeroshot_ger	0.68	0.81	0.74
21	qwen25_7b_4bit_cot_eng	0.77	0.89	0.83
22	qwen25_7b_4bit_cot_ger	0.66	0.88	0.75
23	qwen25_7b_4bit_cov_eng	0.84	0.91	0.87
24	qwen25_7b_4bit_cov_ger	0.71	0.90	0.79
25	qwen25_7b_4bit_fewshot_eng	0.64	0.85	0.73
26	qwen25_7b_4bit_fewshot_ger	0.37	0.82	0.51
27	qwen25_7b_4bit_rephrase_eng	0.75	0.91	0.82
28	qwen25_7b_4bit_rephrase_ger	0.55	0.87	0.68
29	qwen25_7b_4bit_zeroshot_eng	0.82	0.91	0.86
30	qwen25_7b_4bit_zeroshot_ger	0.72	0.90	0.80
